# A Hypothesis for Using Pathway Genetic Load Analysis for Understanding Complex Outcomes in Bilirubin Encephalopathy

**DOI:** 10.3389/fnins.2016.00376

**Published:** 2016-08-18

**Authors:** Sean M. Riordan, Douglas C. Bittel, Jean-Baptiste Le Pichon, Silvia Gazzin, Claudio Tiribelli, Jon F. Watchko, Richard P. Wennberg, Steven M. Shapiro

**Affiliations:** ^1^Division of Child Neurology, Department of Pediatrics, Children's Mercy HospitalKansas City, MO, USA; ^2^Department of Neurology, University of Kansas Medical CenterKansas City, KS, USA; ^3^Ward Family Heart Center, Children's Mercy HospitalKansas City, MO, USA; ^4^Department of Pediatrics, University of Missouri-Kansas City School of MedicineKansas City, MO, USA; ^5^Department of Pediatrics, University of Kansas Medical CenterKansas City, KS, USA; ^6^Italian Liver Foundation, Centro Studi Fegato (CSF)Trieste, Italy; ^7^Department of Medical Sciences, University of TriesteTrieste, Italy; ^8^Division of Newborn Medicine, Department of Pediatrics, University of Pittsburgh School of MedicinePittsburgh, PA, USA; ^9^Department of Pediatrics, University of WashingtonSeattle, WA, USA

**Keywords:** bilirubin, kernicterus, pathway genetic load score, bilirubin encephalopathy, GWAS

## Abstract

Genetic-based susceptibility to bilirubin neurotoxicity and chronic bilirubin encephalopathy (kernicterus) is still poorly understood. Neonatal jaundice affects 60–80% of newborns, and considerable effort goes into preventing this relatively benign condition from escalating into the development of kernicterus making the incidence of this potentially devastating condition very rare in more developed countries. The current understanding of the genetic background of kernicterus is largely comprised of mutations related to alterations of bilirubin production, elimination, or both. Less is known about mutations that may predispose or protect against CNS bilirubin neurotoxicity. The lack of a monogenetic source for this risk of bilirubin neurotoxicity suggests that disease progression is dependent upon an overall decrease in the functionality of one or more essential genetically controlled metabolic pathways. In other words, a “load” is placed on key pathways in the form of multiple genetic variants that combine to create a vulnerable phenotype. The idea of epistatic interactions creating a pathway genetic load (PGL) that affects the response to a specific insult has been previously reported as a PGL score. We hypothesize that the PGL score can be used to investigate whether increased susceptibility to bilirubin-induced CNS damage in neonates is due to a mutational load being placed on key genetic pathways important to the central nervous system's response to bilirubin neurotoxicity. We propose a modification of the PGL score method that replaces the use of a canonical pathway with custom gene lists organized into three tiers with descending levels of evidence combined with the utilization of single nucleotide polymorphism (SNP) causality prediction methods. The PGL score has the potential to explain the genetic background of complex bilirubin induced neurological disorders (BIND) such as kernicterus and could be the key to understanding ranges of outcome severity in complex diseases. We anticipate that this method could be useful for improving the care of jaundiced newborns through its use as an at-risk screen. Importantly, this method would also be useful in uncovering basic knowledge about this and other polygenetic diseases whose genetic source is difficult to discern through traditional means such as a genome-wide association study.

## Introduction

Chronic bilirubin encephalopathy (CBE—also known as kernicterus) is caused by exposure to toxic levels of unconjugated bilirubin (UCB) in severely jaundiced newborns resulting in deposition of bilirubin in the brain (Shapiro, 2010). Neonatal jaundice, present in 60–80% of newborns, is commonly due to unconjugated hyperbilirubinemia and is assessed clinically by measuring total serum bilirubin (TSB) and UCB. Effective monitoring and treatment has helped make the development of CBE a rare event in the majority of countries despite the prevalence of neonatal jaundice. The concentration of albumin, the major binding protein of bilirubin in the blood, and its ability to bind bilirubin are important factors in the transport of bilirubin to the liver for clearance. To prevent excessive hyperbilirubinemia infants are commonly treated with phototherapy to convert bilirubin into water-soluble photoisomers that can be excreted by the liver and kidneys. When hyperbilirubinemia exceeds a specific level defined in clinical guidelines (e.g., American Academy of Pediatrics Subcommittee on Hyperbilirubinemia, 2004) exchange transfusions may be performed. While these treatments have significantly decreased the overall incidence of CBE in most industrialized countries, CBE with lifelong neurological sequelae still occurs, and is a major problem in many areas of the world, especially in low- and middle-income countries (Olusanya et al., 2014; Greco et al., 2016).

Despite the widespread use of TSB levels as a guide for intervention in jaundiced newborns, TSB alone has been shown repeatedly to be a poor predictor of neurological impairment (Ahlfors et al., 2009; Gamaleldin et al., 2011). TSB measures bilirubin levels in blood, where more than 99% of bilirubin is bound to proteins, especially albumin (90% or more) (Hulzebos and Dijk, 2014), but does not measure bilirubin in brain tissue. The toxic form of bilirubin, i.e., free, unbound bilirubin (Bf) correlates better with both acute and chronic bilirubin encephalopathy (Nakamura et al., 1985; Wennberg et al., 2006; Ahlfors et al., 2009; Amin et al., 2016). The inability to easily and reliably measure Bf levels, in addition to the complex nature of unbound bilirubin deposition and toxicity, has impaired investigations of predictive accuracy of Bf for CBE. Furthermore, subtle chronic bilirubin encephalopathy, part of a spectrum of kernicterus or bilirubin-induced neurological dysfunction (BIND) (Shapiro, 2005, 2010; Bhutani and Johnson-Hamerman, 2015), has been difficult to link retrospectively to TSB and UCB levels.

We hypothesize that the production, transport, and elimination of bilirubin, as well as the cellular response to bilirubin, have an important genetic component. If this genetic component could be better understood, it would enhance identification of patients at risk and allow for an individualized therapeutic approach to hyperbilirubinemia. In this paper, we first review genetic methods commonly used to study poly-genetic diseases in large populations and show that these methods cannot be used in a disease such as kernicterus. We then propose a modified use of the pathway genetic load (PGL) score method to decipher the genetic factors that contribute to increased susceptibility to bilirubin neurotoxicity. Finally, we summarize our proposal and describe the current status of this ongoing project.

## The promise of genetics

### Genetic analysis and disease

The study of molecular genetics has identified more than 6000 different monogenetic diseases, mostly through familial linkage analysis. Autosomal dominant, autosomal recessive, and X-linked genetic diseases with complete penetrance were the first and easiest to identify by linkage analysis. Later, genome-wide association studies (GWAS) were developed to identify genes contributing to incompletely penetrant and poly-genetic diseases. Both methods have successfully identified key genetic contributors to various diseases. However, these genetic analyses have also had many failures. Studies of thousands of individuals have failed to uncover significant genetic heritability for numerous diseases (the “missing heritability” problem), leading to a re-evaluation of the basic tenants of GWAS (reviewed in Marjoram et al., 2014).

### Current strategies: successes and shortcomings

Family linkage analysis is very good at identifying rare mutations with large effects that confer susceptibility to common diseases. Examples include the *BRCA1* and *BRCA2* genes in breast cancer (Easton et al., 1995; Ford et al., 1998). By comparison, GWAS studies excel at analyzing complex diseases and disease genes with a weak effect (Risch and Merikangas, 1996). The most common target in GWAS studies has been the single nucleotide polymorphism (SNP) (Palmer and Cardon, 2005). SNPs identified in GWAS studies have largely been shown to be representative of loci of interest and thus have an indirect association with the actual causal variant(s). For example, the *FTO* gene has been shown through GWAS studies to associate with obesity. However, its effect on obesity was eventually shown to be through an enhancer mechanism on downstream genes, including *IRX3* and *IRX5* (Smemo et al., 2014; Claussnitzer et al., 2015). A major advantage of GWAS studies is the unbiased inclusion of the entire genome, thus allowing for the identification of novel loci of interest and the development of original hypotheses to be tested. Once statistically significant associations have been established with one or more loci, they can be confirmed with data from other GWAS populations or through determination of the causal relationship by experimental means (Sekar et al., 2016).

While GWAS studies have produced important discoveries, their experimental designs impose hurdles that can present significant challenges. The first hurdle is population size. GWAS studies are, by definition, very large, requiring scans of large numbers of SNPs. In order to reach statistical significance, sample sizes in the thousands are required. For common diseases, very large sample sizes are attainable; however, for rarer diseases, sample size becomes problematic and is prohibitive in some cases. The second hurdle is that GWAS studies focus on identifying one or sometimes a small group of mutations in loci that are associated with a disease phenotype. It is then left to interpretation what, if any, relationship there is between these loci and the condition being studied. The third hurdle is that GWAS studies largely operate on the common disease–common variant hypothesis. This hypothesis states that the genetic contributions to the susceptibility *of common diseases are attributable to a small number of variants present in more than 1–5% of the population* (Manolio, 2010). While this hypothesis has proven useful for common diseases, it is not as applicable to rare diseases and more complex diseases whose mutations may not reach the 1% threshold. The fourth and final hurdle is that GWAS is not well suited to diseases in which the genetic impact on a disease state is largely dependent upon epistatic interactions. Epistatic interactions are described as the interaction between two loci whereby the phenotype of one locus depends on the genotype of the second locus (Carlborg and Haley, 2004). For quantitative traits, epistasis describes a situation where the sum of the single locus effects cannot be used to predict the phenotype of a particular genotype (Carlborg and Haley, 2004). When considering the effect of genetic interaction at the pathway level, one or two small effect mutations may not cause a noticeable phenotype, but 10 or 20 mutations could combine to create a significant “load” on the pathway and cause deterioration in functionality, leading to a diseased state. Epistatic interactions could also occur within a single gene where one SNP functionally affects another. In this model, neither mutation alone causes an effect, but together they combine to act synergistically to cause an effect greater than would be predicted by an additive model.

## An alternative approach: the pathway genetic load (PGL) score

### Description of the pathway genetic load score

The GWAS method has identified a number of genetic associations that are important to numerous complex diseases. Within these predictions, often a large portion of the heritability remains to be uncovered. Understanding this “missing heritability” has become one of the major goals of clinical genetic research. Some of the tactics that have been proposed include combining linkage and association analysis (Ott et al., 2011), correcting an overestimation in heritability (Zuk et al., 2012), and considering gene–gene interactions (Liu et al., 2012).

A particularly intriguing approach to resolving the missing heritability problem was recently presented by Huebinger et al., who used a PGL risk score (Huebinger et al., 2010) to predict the outcome of patients exposed to severe burn trauma. The PGL risk score was defined as the “sum total of mutant alleles present at risk loci within a common biological pathway.” The PGL risk score is calculated using a number of assumptions that require certain concessions to be made in order to be both useful and accurate. Others have used similar pathway analysis methods to study genetic risks for coronary infarction (Yiannakouris et al., 2006; Trichopoulou et al., 2008) and Parkinson's disease (Lesnick et al., 2007). The PGL method has the potential to offer an alternative method to GWAS for identifying relevant genetic contributors, especially for lower incidence diseases.

## Applying the PGL risk score to bilirubin encephalopathy

### Description of bilirubin encephalopathy

Kernicterus or CBE is a rare disease in affluent countries because of preventive measures. However, neonatal jaundice is very common, occurring in 60–80% of full term (≥37 weeks' gestational age) otherwise healthy newborns. About 7% of jaundiced infants receive phototherapy (Bhutani et al., 2004a,b, 2013; Keren et al., 2009). Classical kernicterus is a well-described clinical tetrad of (i) abnormal motor control, movements, and muscle tone, (ii) an auditory processing disturbance with or without hearing loss, (iii) oculomotor impairments, especially impairment of upward vertical gaze, and (iv) dysplasia of the enamel of deciduous (baby) teeth, an abnormality leading to pitting, flaking, and chipping of the enamel (Shapiro et al., 2006). In addition to classical kernicterus, subclasses of kernicterus are characterized as auditory predominant, motor predominant, and subtle kernicterus (BIND) (Shapiro, 2010).

While efforts to prevent dangerous hyperbilirubinemia have been successful in the developed world, a high incidence of glucose-6-phosphate dehydrogenase deficiency, delays in seeking care, lack of TSB screening resources, and ineffective treatments in low and middle income countries ensure that bilirubin encephalopathy remains a serious world health problem (Olusanya et al., 2014; Slusher et al., 2015). Ogunlesi et al. (2007) reported a 2.7% incidence of bilirubin encephalopathy among neonates admitted to two hospitals in Nigeria between 2002 and 2005. This high prevalence of kernicterus, in conjunction with new reports of the more subtle predominantly auditory and predominantly motor forms of kernicterus (Shapiro and Popelka, 2011), underscores the need to better understand why hyperbilirubinemia causes permanent brain damage in some infants and not in others. The relative rarity of kernicterus in the West and, to a lesser extent, milder forms of bilirubin encephalopathy, makes it exceedingly difficult to perform traditional genetic analysis through large population GWAS studies. In addition, the apparent wide range of susceptibility to a common bilirubin exposure points toward polygenetic determination of risk for neurotoxicity. The current understanding of the genetics of bilirubin encephalopathy (described below) also points toward a polygenetic cause for increased risks to bilirubin neurotoxicity.

Together, these factors indicate a need for an alternative method, such as the PGL risk score, to understand genetic factors contributing to risk for CBE. The PGL method is a good fit for bilirubin encephalopathy because bilirubin encephalopathy results from a one-time insult, similar to the severe burns examined in Huebinger et al.'s study. For bilirubin encephalopathy that insult is in the form of moderate to extreme hyperbilirubinemia at birth. Also, similar to the total body surface area measure for a serious burn, the body's response to bilirubin toxicity is variable and not well explained by its key diagnostic test, peak TSB levels.

While significant progress has been made in understanding the various causes of neonatal hyperbilirubinemia, few studies have been undertaken to understand the mechanism of bilirubin toxicity. Multiple cellular and molecular cascades likely underlie bilirubin-induced neuronal injury, including plasma membrane perturbations, excitotoxicity, neuroinflammation, oxidative stress, and cell cycle arrest. It is likely that variable effectiveness of the cellular response to bilirubin exposure contributes to the range of observed clinical outcomes. To explain part of this variability, we hypothesize that a combination of SNPs in key pathways are responsible for creating a vulnerable state to bilirubin neurotoxicity. The development of this vulnerable state likely occurs through a combination of increased CNS bilirubin levels and impaired cellular response to bilirubin.

On the basis of the current understanding of bilirubin encephalopathy, we suspect that the genetic impact is the result of the combined effect of mutations with relatively small effects that, if present alone, would otherwise be clinically benign. Alternative hypotheses that could also explain the TSB's poor predictive value for kernicterus include disrupted albumin/bilirubin binding capacity, reductions in available albumin, and use of TSB levels as the sole diagnostic test for bilirubin exposure. A number of studies have been published that examine the issue of TSB's reliability in both predicting and improving outcomes. These studies have compared the effectiveness of alternatives to TSB such as albumin/bilirubin binding capacity (Kapitulnik et al., 1974), unbound (free) bilirubin levels (Cashore and Oh, 1982; Ahlfors et al., 2009; Morioka et al., 2015), and bilirubin-albumin ratios either in place of or in addition to TSB testing (Ahlfors, 1994; Amin et al., 2001, 2016).

While Nakamura et al. showed over 30 years ago that free bilirubin levels predict neurotoxicity better than TSB levels do (Nakamura et al., 1985), technical issues, reliability, and lack of availability of these tests have called their usefulness into question (McDonagh and Maisels, 2006; Hulzebos and Dijk, 2014). The use of the bilirubin/albumin ratio has also been shown to be as strong a predictor of neurotoxicity as TSB (Iskander et al., 2014) but has not been shown to offer an advantage in predicting or improving outcomes over TSB alone (Iskander et al., 2014) or when used in addition to TSB (Hulzebos et al., 2014).

### Known genetics of bilirubin encephalopathy

Bilirubin is largely a breakdown product of hemoglobin. Bilirubin rises after birth in all mammals, and in humans usually peaks at 3–5 days after birth and then falls to very low values through the rest of life (Kivlahan and James, 1984; Maisels et al., 2014). It is bound in the blood to proteins, especially albumin. Very little bilirubin normally reaches brain tissue. There is evidence that at low levels bilirubin is beneficial, acting as a natural antioxidant (Adin et al., 2005; Bakrania et al., 2016), but very high levels can exceed the buffering capacity of the blood and allow bilirubin to move into the brain, causing a unique pattern of brain injury (Shapiro et al., 2006; Shapiro, 2010).

Hyperbilirubinemia results from the excessive breakdown of red blood cells (RBC) (hemolysis) or impaired elimination of bilirubin. Most genetic abnormalities linked to the presentation of bilirubin encephalopathy are related to production and elimination. Hemolysis may occur as a result of immune mechanisms (e.g., Rh disease, ABO blood group incompatibilities), increased fragility of RBC [Glucose-6-phosphate dehydrogenase (G6PD) deficiency], or from bleeding (e.g., cephalohematoma). Decreased elimination is enhanced in infants with genetic variations in the enzyme or the promotor regions of UDP-glucuronosyltransferase 1A1 (*UGT1A1*), (Watchko and Tiribelli, 2013). Other factors that contribute to hyperbilirubinemia include decreased oral intake and stooling. It is believed that hyperbilirubinemia due to decreased oral intake may account for a substantial number of readmissions of newborns to hospitals after discharge (Seidman et al., 1995; Farhat and Rajab, 2011; Maisels et al., 2014).

### Outcome variability at the same or very similar TSB levels

Possible sources of outcome variability include factors such as the source of hyperbilirubinemia, the gestational age of the infant, the duration of exposure, the kinetics of bilirubin's movement between miscible compartments of the body (e.g., blood, tissue, CNS, extracellular, and intracellular), the type of treatment administered and other known risk factors such as acidosis or sepsis (Shapiro, 2010; Hulzebos and Dijk, 2014). Furthermore, a significant genetic contribution to the susceptibility of an individual to neurological damage due to hyperbilirubinemia may be related to the disruption of mitochondria, which may in turn affect cellular and molecular cascades including plasma membrane perturbations, excitotoxicity, neuroinflammation, oxidative stress, and cell cycle arrest referred to previously (Rodrigues et al., 2002a,b; Vaz et al., 2010; Brites, 2011; Barateiro et al., 2012).

Clinical observations have shown that in cases where TSB reaches extremely high levels (>40 mg/dL or >680 μM^1^), the likelihood of developing at least some form of kernicterus approaches 100%, although exceptions to this generalization have been published (Newman et al., 2003, 2006). These clinical observations likely indicate that for most neonates, there is a maximum level of bilirubin or “tipping point” where the body's natural defense mechanisms for clearing unbound bilirubin from susceptible neurons are overwhelmed and deposition occurs. However, because kernicterus has been known to develop at lower TSB levels, it is clear that the maximum level of bilirubin exposure varies from person to person (Ritter et al., 1982; Odutolu and Emmerson, 2013). Even allowing for external factors and the inexact nature of TSB as a measure of exposure to bilirubin in brain tissue, there is still a strong likelihood that genetic variability contributes to the overall susceptibility of a child to bilirubin neurotoxicity and subsequent chronic adverse neurodevelopmental sequelae.

### Selecting a “pathway” for bilirubin encephalopathy

Here we propose using the PGL method in order to determine the extent to which a multifactorial genetic response contributes to the variable susceptibility to bilirubin toxicity and the resultant variability in disease outcome. Due to the lack of a CBE GWAS to guide our pathway selection, we propose creating three custom “pathways” of genes that are organized into “tiers” based on the mechanism of physiological contributions and the relative strength of evidence that these genes contribute to bilirubin susceptibility and sensitivity. Each of these three gene tiers will be analyzed individually and independently using the PGL method.

The PGL method differs from traditional association analysis in a number of important ways. First, the PGL score uses a biased approach by leveraging what is already known about a disease to focus on a specific set of genes (pathway) and SNPs. Second, unlike GWAS and linkage analysis, the PGL score method involves the calculation of a risk score. Third, the method can be performed using a smaller sample size than in GWAS studies because the number of variables is dependent on candidate pathway size, thus reducing the required sample size to achieve adequate statistical power. Finally, the PGL method allows detection of combinatorial epistatic interactions that would otherwise have been missed by both GWAS and linkage analysis.

The decision to use a biased approach comes with the understanding that the available knowledge will very likely present only a partial picture of the entire genetic heritability. However, the hope is that if enough of the biological background is known, then the analysis will prove to be useful and actionable. By accepting a higher rate of false negatives, we will find it is then possible to drastically reduce the number of genes and thus variants being surveyed. Reducing the number of SNPs being investigated allows for a corresponding reduction in sample size, opening up analysis to rarer diseases. Huebinger et al. used their knowledge of injury and infection to select the Toll-like receptor signaling pathway to assess whether an increased PGL risk score within this pathway could predict the outcome following burn trauma (Huebinger et al., 2010). Similarly, using the PGL method, we will perform a targeted genetic analysis on a small number of samples and will determine whether a common mutational “load” exists in our affected individuals that points toward the development of a state of bilirubin hypersensitivity.

#### Tier 1: known genetic risk factors for increased hyperbilirubinemia: genes for increased production or decreased elimination of bilirubin

The tier 1 “pathway” is composed of genes known as contributing risk factors for hyperbilirubinemia and bilirubin encephalopathy (i.e., classically recognized) (Watchko and Lin, 2010, 2012; Watchko, 2013). This pathway includes all of the known mechanisms contributing to the production and elimination of bilirubin.

The production pathway includes genes that predispose to hemolysis or reduce RBC lifespan, leading to increased bilirubin production in neonates. *G6PD* and *Pyruvate Kinase (PK)* mutations can cause defects in RBC metabolism (Watchko and Lin, 2012). Several conditions lead to reduced RBC lifespan such as hereditary spherocytosis, elliptocytosis, stomatocytosis, and infantile pyknocytosis (Delaunay, 2007; Kraus et al., 2010; Kaplan et al., 2014).

Genes that affect elimination of bilirubin include allelic variation in the regulatory region of the *UGT1A1* gene (affecting bilirubin conjugation in the liver, Gilbert's syndrome) and the solute transporter *SLCO1B1* that facilitates UCB uptake and influences the ability to clear bilirubin. Poorly functioning variants of either gene can contribute to hyperbilirubinemia (Johnson et al., 2009; Watchko and Lin, 2010). We consider these genes to be the first tier of primary candidate genes that are known to influence bilirubin levels. It is reasonable to presume that a combination of subtle, seemingly benign mutations in these genes could result in increasing bilirubin to toxic levels. The complete tier 1 list can be seen in (Supplemental Table 1).

#### Tier 2: *in vitro*—genetic responses to bilirubin in neuronal and glial cell cultures

The tier 2 “pathway” is composed of genes identified in recent studies as genes whose expression levels are modulated by bilirubin exposure. These genes were identified by *in vitro* experiments in human SH-SY5Y neuroblastoma cells (Calligaris et al., 2009). In these experiments, the cells were exposed to UCB for 24 h and transcriptome changes were examined by high-density oligonucleotide microarrays. Selected genes were then validated by RT-PCR. These genes represent neuronal genes that had altered expression in response to bilirubin exposure. In addition, we have also included those genes that are related to the immunoreactive response shown to occur in primary rodent neuronal and glial cultures exposed to UCB (Brites, 2012). Primary glial cell lines including rat astrocytes (Fernandes et al., 2004, 2006, 2007b) rat microglia (Gordo et al., 2006; Silva et al., 2010), and rat oligodendrocytes (Barateiro et al., 2012, 2013, 2014) have been shown to be sensitive to exposure of UCB leading to cell death in all cases. In addition, rat astrocytes and microglia, but not oligodendrocytes release pro-inflammatory cytokines upon exposure to UCB (Gordo et al., 2006; Fernandes et al., 2007a; Barateiro et al., 2012). It is thought that this inflammatory response is a crucial element in the UCB response and plays a major role in the cell death (Vaz et al., 2011; Silva et al., 2012). Unlike the list of genes in tier 1, this list represents a response to bilirubin exposure rather than the production of a hyperbilirubinemic state. We hypothesize that this list contains a core network of genes that are important at the cellular level to respond to toxic levels of bilirubin. The complete tier 2 list can be seen in Supplemental Table 2.

#### Tier 3: *in silico*—genes identified by bioinformatics analysis

We hypothesize that gene expression differences in susceptible vs. resistant regions of the brain may provide some insight into additional secondary mediators of bilirubin neurotoxicity. The tier 3 “pathway” is composed of genes identified to be differentially expressed in regions of the brain that are susceptible to bilirubin compared to similar regions in close proximity that are resistant. We chose two pairs of susceptible vs. resistant brain regions for comparison based on clinical knowledge (Shapiro, 2005, 2010) and animal studies (Gazzin et al., 2012): (1) the inferior colliculus (IC) is susceptible to bilirubin toxicity compared to, superior colliculus (SC), the resistant control, and (2) the globus pallidus (GP) vs. putamen (PU). The IC is in part responsible for the auditory abnormalities and the GP for the motor abnormalities in kernicterus and BIND. Furthermore, in addition to showing a marked difference in response to bilirubin toxicity, these regions of the brain are in close proximity to each other, thus minimizing the potential for variability in blood flow and other factors.

The Allen Brain Atlas is a collection of freely available online resources integrating extensive gene expression and neuroanatomical data from developing and adult human brains (Website: © 2015 Allen Institute for Brain Science. Allen Human Brain Atlas [Internet]. Available from: http://human.brain-map.org) (Hawrylycz et al., 2012). The atlas allows exploration of the expression of known genes in carefully defined anatomical regions of the human brain at high resolution. We used the brain atlas to compare gene expression between regions that are susceptible to bilirubin toxicity and those that are resistant.

For the creation of Tier 3 A and B we identified those genes whose expression was significantly different (*p* < 0.05) between the IC (susceptible) compared to SC (resistant) brain regions in normal adult brains using the resources of the Allen Brain Atlas. From these genes we selected 100 genes with the largest increase in expression level in the IC vs. SC (3A) and 100 genes with the largest increase in expression level in the SC vs. IC (3B), as measured by fold change, for a total of 200 genes. For the creation of Tier 3 C and D we identified those genes whose expression was significantly different (*p* < 0.05) between GP (susceptible) and PU (resistant) brain regions. And as with 3A and 3B we selected 100 genes with the largest increase in expression level in the GP vs. PU, (3C) and 100 genes with the largest increase in expression level in the PU vs. GP (3D), as measured by fold change, for a total of 200 genes.

We chose to limit our gene lists to 200 genes per comparison because we believe that this strategy provides a fair representation of those genes that show the most dramatic change between regions and thus potentially reflect the genetic components underlying the physiological differences that contribute to region specific bilirubin toxicity resistance or susceptibility. While we recognize that this strategy will not encompass all the genes responsible for the varied response to bilirubin we believe that this abbreviated strategy will allow for the identification of major contributors and their associated pathways thus opening up new avenues for future research. We hypothesize that analyzing the expression of the genes in these differentially affected regions of the brain will reveal susceptible and protected genetic profiles within the brain that could then be extrapolated to better understand the variable susceptibility of individuals to bilirubin toxicity. The complete tier 3 list can be seen in Supplemental Table 3.

### Selecting key SNPs for PGL risk score calculation and analysis

**Figure 1:**
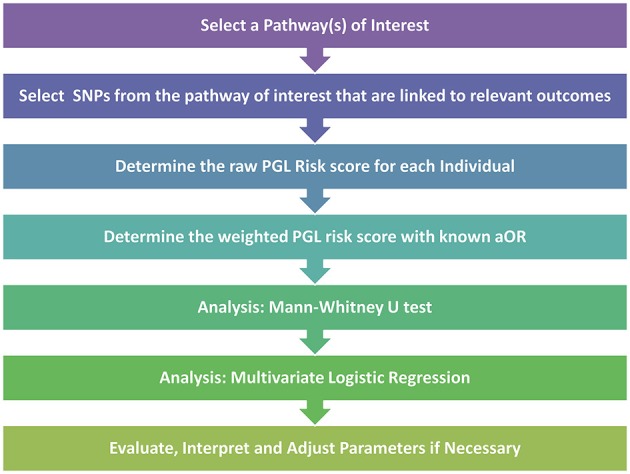
**Pathway genetic load risk score workflow**. Workflow for developing and testing a PGL risk score according to Huebinger et al. (2010). The raw and weighted scores are analyzed separately to assess the impact of relevant disease association data on the PGL risk score calculation. Abbreviations: SNP, Single Nucleotide Polymorphism; PGL, Pathway Genetic Load; aOR, adjusted odds ratio gathered from a relevant GWAS study.

After the pathway of interest is selected, the next step is to select the candidate SNPs from within the pathway. For this crucial step, Huebinger et al. chose to create their candidate SNP list from variants that had previously published associations with burn trauma. Once these SNPs are determined, the PGL risk score can be calculated by summing the numerical scores for genotypes across all SNPs that are homozygous protective (assigned a score of “0”), heterozygous (score of “1”) and homozygous detrimental (score of “2”). The initial calculation results in the raw risk score where each SNP is treated equally. The risk score is then weighted to better represent the impact of each of the SNPs. For this step Huebinger et al. chose to use the adjusted odds ratio from previously reported studies for each SNP between each individual locus and either death or sepsis. For loci without previously published data, the value was left unweighted. The data could then be analyzed further by Mann-Whitney U Test to investigate whether the PGL risk score alone can predict developing sepsis. In addition, the multivariate logistic regression test could be used to investigate the simultaneous effect of multiple variables as risk factors for various outcomes. A synopsis of the PGL workflow can be seen in Figure 1.

For their analysis Huebinger et al. chose to adjust for age, gender, race, total body surface area burned, and inhalation injury. They were able to show that each unit increase in weighted PGL risk scores was associated with increased probability of both complicated sepsis and death. By analyzing data from only 155 burn trauma patients and using only 6 SNPs at 6 different loci, they showed that a larger PGL risk score indicated increased odds of both sepsis and mortality in response to burn trauma. This work, along with other similar pathway load work (Lesnick et al., 2007), demonstrates that by using a targeted approach it is possible to show that a genetic load placed on a crucial pathway can have predictive capabilities even with a relatively small sample size.

To develop a SNP set for each tier, we will collect whole exome sequence data from patients with moderate to high TSB levels (10–25 mg/dL and >25 mg/dL respectively) both with and without evidence of classic auditory or motor dysfunction. We will identify positive “hits” through a pipeline utilizing the Partek Genomics Suite and Flow (Partek Inc., St. Louis, MO, USA). Causal variants in this study will then be identified through the use of QIAGEN's Ingenuity® Variant Analysis™ software (www.qiagen.com/ingenuity) from QIAGEN Redwood City. Ingenuity® Variant Analysis™ allows for multiple types of SNP filtering to create a list of screened causal variants that are present in a significant number of the case group vs. controls. The causal variant filter is based on the American College of Medical Genetics and Genomics (ACMG) categorization guidelines (Richards et al., 2015). Alternatively, or in combination with ACMG categorization, it is possible to select variants that cause a gain of function or a loss of function based on information in the Ingenuity® Knowledge Base. The Ingenuity® Knowledge Base is a “repository of expertly curated biological interactions and functional annotations created from millions of individually modeled relationships between proteins, genes, complexes, cells, tissues, drugs, and diseases” manually created from the literature (http://www.ingenuity.com/science/knowledge-base). The causal variant list can also be filtered to include only those genes present in each of the gene set tiers.

Once the key SNPs predicted to be deleterious are identified, the PGL risk score can be calculated. The raw score will be calculated from the individual genotypes as described above as homozygous protective = 0, heterozygous = 1 and compound heterozygote/homozygous deleterious = 2. The raw score will then be summed and the adjusted odds ratio, confidence interval, and *p*-value will be calculated. Multivariate logistic regression will then be used to evaluate the simultaneous effects of multiple variables as risk factors for the development of one of three outcomes. Possible outcomes include predominantly auditory dysfunction, predominantly motor dysfunction, and auditory plus motor dysfunction (classic kernicterus) (Shapiro, 2010). Outcomes will be further analyzed with adjustments for TSB, albumin, gender, race, sepsis, and gestational age at birth.

**Figure 2:**
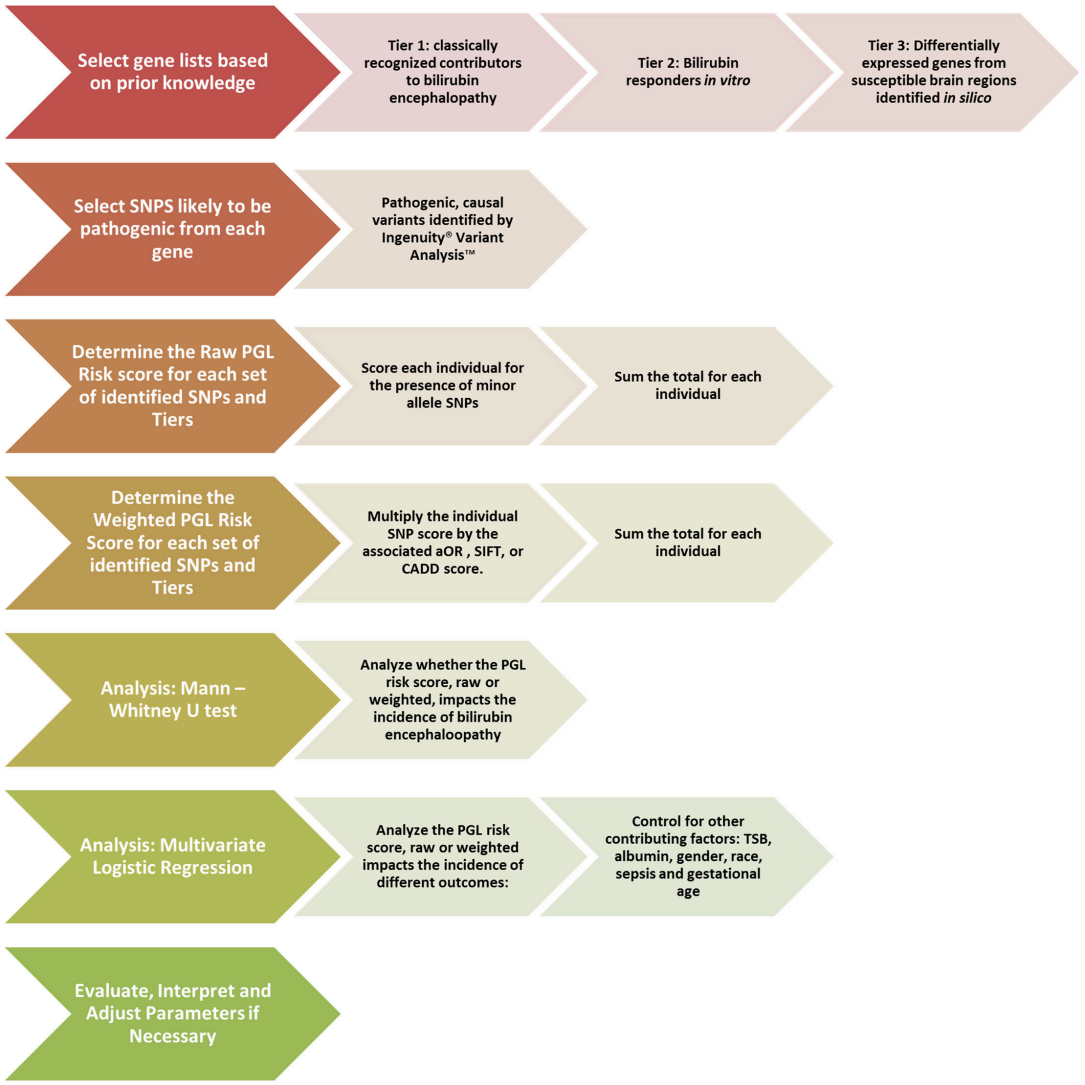
**Proposed pathway genetic load risk score workflow for bilirubin encephalopathy**. The chevrons at each step include a description of the proposed alterations to be made to the original workflow presented in Figure 1. Significant changes were made at the gene set creation, SNP selection and the weighting steps. The analysis steps are largely unchanged. Abbreviations: SNP, Single Nucleotide Polymorphism; PGL, Pathway Genetic Load; aOR, adjusted odds ratio gathered from a relevant GWAS study; SIFT, Sorting Intolerant From Tolerant score; CADD, Combined Annotation Dependent Deletion; TSB, Total Serum Bilirubin.

For weighted analysis, the SNPs will be weighted based on the Sorting Intolerant from Tolerant (SIFT) score (Ng, 2003) or the Combined Annotation-Dependent Deletion (CADD) score (Kircher et al., 2014). By utilizing the ACMG score for initial selection and the SIFT or CADD scores for weighting, we are able to evaluate the direct effect of a SNP, a crucial difference from the GWAS based method. A synopsis of the proposed PGL workflow to be used for bilirubin encephalopathy can be seen in Figure 2.

## Shortcomings of the pathway genetic load score

While the PGL method presented has been successful at producing significant and meaningful results, there are drawbacks to using PGL risk score analysis. These drawbacks include the previously described risk for false negatives, the possibility that major contributing factors are outside the pathway chosen to be studied, and that considerable cross-talk is occurring between overlapping pathways, leading to confusing and difficult to confirm results. In addition, the selections of the target pathway and the set of SNPs are based on the information available at the time of the study which may not be complete enough to make accurate selections. To address this issue, we have used three different methods to develop our tiers aimed at maximizing the currently available knowledge base for CBE. Finally, the weighting method described by Huebinger et al. utilizes an adjusted odds ratio based on previously performed GWAS studies (Huebinger et al., 2010). This type of GWAS data may not be available for rare diseases. To address this issue, we have proposed a method that will weigh the PGL risk score of each SNP using pathogenicity scores that are available from various bioinformatics sources as an attempt to investigate a direct impact of each SNP on the functionality of the pathway of interest.

## Moving forward

To test the method described above, we plan on analyzing whole exome sequence data from patients exposed to moderate (15–25 mg/dL) and high (>25 mg/dL) peak TSB levels at birth. We acknowledge that it would be preferable to also investigate the free unbound bilirubin levels for these patients, but those data are rarely available. Because of the rarity of kernicterus in developed countries, 1.15:100,000 live births in an average of three studies from Denmark (Ebbesen, 2000; Ebbesen et al., 2005; Bjerre et al., 2008) and 0.93:100,000 live births in the United Kingdom and Ireland (Manning et al., 2007), it will likely be necessary to obtain biological samples from international locations such as Africa and southeast Asia where kernicterus, unfortunately, continues to be a significant problem. The inclusion of such disparate populations has the potential to add additional complicating factors, including differences in genetic background and differences in common treatment methods. We will address these issues by comparing patients of similar ethnic backgrounds whenever possible. The collection of biological material for this project has received institutional review board approval and is ongoing. Collaborating with other laboratories internationally is also in progress.

## Summary

We present a method for addressing the problems associated with identifying the genetic factors that influence both level of bilirubin and susceptibility to bilirubin encephalopathy (kernicterus). Because rare diseases by definition affect small numbers of people, it is impractical to use unbiased population-based GWAS studies. In this paper, we present an evidence-based candidate pathway approach in the form of the PGL risk score that will reduce the number of genes and variants investigated and thus reduce the need for overly large sample sizes. We propose new alternatives for selecting pathways and variants through the creation of custom gene lists and use of functional scoring methods. The logistic regression analysis proposed in Huebinger et al. will be used to determine whether the PGL score for patients with moderate and high TSB levels could successfully predict auditory or motor deficit outcomes. As this method is tested and refined, we believe it will lead to the creation of new hypotheses that will aid the investigation of the molecular mechanisms of bilirubin toxicity in newborns. This work could also potentially be used to develop a screening process for jaundiced infants that could inform physicians about whether more aggressive treatment is needed in infants with moderate TSB levels. Finally, this method could be adapted to other rare or complex diseases to address the challenge of missing heritability that exists despite GWAS.

## Author contributions

SR drafted the manuscript. SR, DB, JL, SG, CT, JW, RW, and SS contributed to the conception of the work and provided critical revision for important intellectual content.

## Funding

This work was supported by institutional startup funds from Children's Mercy Hospital.

### Conflict of interest statement

The authors declare that the research was conducted in the absence of any commercial or financial relationships that could be construed as a potential conflict of interest.

## Footnotes

^1^Conversion from international units of bilirubin (μM) to mg/dL (a.k.a. mg%) is 17.1, i.e., 1 mg/dL = 17.1 μM.

## Supplementary material

Supplementary Table 1 **Tier 1: Known genetic risk factors for increased hyperbilirubinemia**. This list represents the classical list of genes associated with hyperbilirubinemia, jaundice and kernicterus. A reference for each gene is listed in the final column.

Supplementary Table 2 **Tier 2: ***In vitro***—genetic responses to bilirubin in neuronal and glial cell cultures**. This list represents the genes reported to be affected in either expression level or protein response in cultured cells exposed. Cell types include human neuronal (SH-SY5Y cells), primary rat astrocytes, primary rat oligodendrocytes and primary rat microglia. A reference for each gene is listed in the final column.

Supplementary Table 3 **Tier 3: ***In silico***—comparative analysis of gene expression in susceptible and resistant brain regions in data from the Allen Brain Atlas**. Gene expression data from the Allen Brain Atlas (*n* = 6) was collected from two brain region comparisons. 3A shows significantly different (*p* < 0.05) genes with the 100 largest fold change differences that were upregulated in the IC vs. SC. 3B shows significantly different (*p* < 0.05) genes with the 100 largest fold change differences that were upregulated in the SC vs. IC. 3C shows significantly different (*p* < 0.05) genes with the 100 largest fold change differences that were upregulated in the GP vs. PU. 3D shows significantly different (*p* < 0.05) genes with the 100 largest fold change differences that were upregulated in the PU vs. GP. This list was last updated on 7/14/16.
